# *Calotropis gigantea* extract induces apoptosis through extrinsic/intrinsic pathways and reactive oxygen species generation in A549 and NCI-H1299 non-small cell lung cancer cells

**DOI:** 10.1186/s12906-019-2561-1

**Published:** 2019-06-18

**Authors:** Jiyon Lee, Hui-Ju Jang, Hyunwoo Chun, Thu-Huyen Pham, Yesol Bak, Jong-Woon Shin, Hang Jin, Yong-In Kim, Hyung Won Ryu, Sei Ryang Oh, Do-Young Yoon

**Affiliations:** 1 Laboratory of Cell Biology and Immuno-biochemistry, Department of Bioscience and Biotechnology, Konkuk University, 120 Neungdong-ro, Gwangjin-gu, Seoul, 05029 Republic of Korea; 2 Institute of Medicinal Plants, Yunnan Academy of Agricultural Sciences (YAAS), 2238 Beijing Road, Kunming, 650205 Yunnan Province China; 3 International Biological Material Research Center, Korea Research Institute of Bioscience and Biotechnology, 125 Kuahak-ro, Youseong-gu, Daejeon, 34141 South Korea; 4 Natural Medicine Research Center, Korea Research Institute of Bioscience and Biotechnology, 30 Yeongudanji-ro, Ohsong, Cheongju, 28116 South Korea; 5 Department of Bioscience and Biotechnology, Konkuk University, 120 Neungdong-ro, Jayang-dong, Gwangjin-gu, Seoul, 05029 Republic of Korea

**Keywords:** *Calotropis gigantea*, Non-small cell lung cancer cell, Anti-cancer, Apoptosis, ROS

## Abstract

**Background:**

*Calotropis gigantea* (CG) is a tall and waxy flower that is used as a traditional remedy for fever, indigestion, rheumatism, leprosy, and leukoderma. However, the precise mechanisms of its anticancer effects have not yet been examined in human non-small cell lung cancer (NSCLC) cells. In this study, we investigated whether CG extract exerted an apoptotic effect in A549 and NCI-H1299 NSCLC cells.

**Methods:**

The ethanol extract of CG was prepared, and its apoptotic effects on A549 and NCI-H1299 NSCLC cells were assessed by using the 3-(4,5-dimethylthiazol-2-yl)-5-(3-carboxy methoxyphenyl)-2-(4-sulfophenyl)-2H-tetrazolium (MTS) assay, annexin V-fluorescein isothiocyanate/propidium iodide (PI) staining, cell cycle analysis, real-time polymerase chain reaction (RT-PCR), western blotting, JC-1 staining, and ROS detection assay.

**Results:**

The CG extract induced apoptosis through the stimulation of intrinsic and extrinsic signaling pathways in A549 and NCI-H1299 lung cancer cells. Cell cycle arrest was induced by the CG extract in both cell lines. Reactive oxygen species (ROS), which can induce cell death, were also generated in the CG-treated A549 and NCI-H1299 cells.

**Conclusions:**

These data confirmed that CG caused apoptosis through the activation of extrinsic and intrinsic pathways, cell cycle arrest, and ROS generation in A549 and NCI-H1299 lung cancer cells. Thus, CG can be suggested as a potential agent for lung cancer therapy.

**Electronic supplementary material:**

The online version of this article (10.1186/s12906-019-2561-1) contains supplementary material, which is available to authorized users.

## Background

Lung cancer, also known as lung carcinoma, is one of the most common diseases in the world [1]. However, as only a few therapies are available, a diverse range of studies on lung cancer are needed. Lung cancer is classified into non-small cell lung cancers (NSCLC) and small cell lung cancers (SCLC) [2, 3]. SCLC is a type of neuroendocrine tumor, and the size of the cells in these cancers is smaller than those in NSCLC. NSCLCs include squamous cell carcinomas, large cell carcinomas, and adenocarcinomas; p53 wild-type A549 cells are human alveolar basal epithelial adenocarcinoma cells and p53 null NCI-H1299 cells are human epithelial carcinoma cells [4]. Lung cancer is caused by uncontrolled cell growth in the lung tissues due to defects in cancer suppressor genes [5] that result in the failure of apoptotic signaling.

Apoptosis is a process of programmed cell death that controls cell proliferation [6]. Cell cycle disruption is a major cause of apoptosis in lung cancer cells [7], as many factors, including p53, p27, p21, and cyclins, control the phases of the cell cycle. In addition, there are two major apoptotic pathways: the intrinsic pathway and the extrinsic pathway [8]. Initially, the intrinsic apoptotic pathway begins when the mitochondrial outer membrane becomes permeable, which may occur in response to intracellular stresses, such as DNA damage, growth factor impairment, or oncogene activation [9]. Whereas, the extrinsic apoptotic pathway is triggered by the death receptor and ligand following the caspase-8 dependent signaling cascade. Finally, both apoptotic pathways induce the inactivation of poly (ADP-ribose) polymerase (PARP), whose function is DNA damage repair [10], to destroy the cells [11].

Programmed cell death can also occur through the initiation of various types of stress-induced damage. Reactive oxygen species (ROS) production [12] is a critical stressor that causes cell death, especially through the induction of apoptosis [13]. The products of ROS generation, such as superoxide (O_2_^−^), hydrogen peroxide (H_2_O_2_), and hydroxyl radicals (^•^OH), initiated by various external stimuli, are related to the inhibition of cell proliferation [14]. The reaction of superoxide, a precursor of ROS generated by mitochondrial electron transport chain activity, to hydrogen peroxide is catalyzed by superoxide dismutase 2 (SOD2), an enzyme that mitigates ROS in the mitochondria [15]. Another antioxidant enzyme, catalase, catalyzes the formation of water (H_2_O) from hydrogen peroxide [16]. However, a low expression of antioxidant enzymes, such as SOD2 and catalase, induces the generation of ROS, which cause in cell death [17]. Furthermore, mitochondria-related proteins, such as B-cell leukemia/lymphoma 2 (Bcl-2) and Bcl-2-associated X protein (Bax), not only control intrinsic apoptotic death, but also the antioxidant pathway [18].

*Calotropis gigantea* (CG) is a tall and waxy flower that is mainly distributed throughout Asia and tropical Africa. The plant is used as a traditional remedy for fever, indigestion, rheumatism, leprosy, and leukoderma [19]. Although the anticancer effects of CG have been reported in colon cancer cells [20, 21], the precise anti-cancer mechanisms of CG have not been elucidated in human lung cancer cells. Here, we have shown that CG extract induces apoptosis via the extrinsic and intrinsic pathways and ROS generation in p53 wild-type A549 and p53 null-type NCI-H1299 NSCLC cells.

## Methods

### Reagents and antibodies

CG was dissolved in 0.05% dimethyl sulfoxide (DMSO) and used for biological assays. CellTiter 96® AQueous One Solution Cell Proliferation Assay Reagent [MTS; 3-(4,5-dimethylthiazol-2-yl)-5-(3-carboxymethoxyphenyl)-2-(4-sulfophenyl)-2H-tetrazolium] was purchased from Promega (Madison, WI, USA), and propidium iodide (PI) was purchased from Sigma-Aldrich (St. Louis, MO, USA). Antibodies specific to PARP, caspase-3, caspase-8, caspase-9, Bcl-2, Bcl-xL, Bax, Bid, and cytochrome c were sourced from Cell Signaling Technology (Beverly, MA, USA). Anti-rabbit IgG horseradish peroxidase (HRP)-conjugated secondary antibody and anti-mouse IgG HRP-conjugated secondary antibody were obtained from Millipore (Billerica, MA, USA). Antibodies specific to p21, p27, cyclin D1, cyclin E, cyclin A, SOD-2, and glyceraldehyde 3-phosphate dehydrogenase (GAPDH) were purchased from Santa Cruz Biotechnology (Santa Cruz, CA, USA). JC-1 (5,5′,6,6′-tetrachloro-1,1′,3,3′-tetraethyl benzimidazoly carbocyanine chloride) was obtained from Enzo (New York, USA), FITC-annexin V apoptosis detection kit I was obtained from BD Biosciences (San Diego, CA, USA), and 2′,7′-dichlorofluorescin diacetate (DCF-DA) was procured from Abcam (Cambridge, UK).

### Plant material and preparation

The ethanol extract of the whole plant of *C. gigantea* (L.) W.T. Aiton (*Asclepiadaceae*) was supplied by Foreign Plant Extract Bank (No. FBM085–042; Daejeon, Korea). The plant was collected in Yunnan Province of China in 2008 and authenticated by Jin Hang, the Chief of the Medicinal Plants Research Institute, Yunnan Academy of Agricultural Sciences (YAAS) (Yunnan, China). A voucher specimen (YASS3533–2) was deposited at the herbarium of YAAS. To prepare the material, the air-dried whole plant of the *C. gigantea* sample (100.0 g) was mixed in 95% ethanol (800 mL × 2), and the mixture was shaken at room temperature for 2 h. The extracts were combined and concentrated in vacuo at 40 °C to produce a dried extract, which then was used for phytochemical analysis and biological assays.

### UPLC-QTof-MS analysis

Tentative identification of compounds from *C. gigantea* extracts were carried out using an ACQUITY UPLC (Waters Corporation, Milford, MA) system connected to a Micromass QTof Premier™ mass spectrometer (Waters Corporation, Milford, MA) with an electrospray ionization device. The operation parameters used in the negative ion mode were: capillary voltage, 2300 V; cone voltage, 50 V; ion source temperature, 110 °C; desolvation temperature, 350 °C; flow rate of desolvation gas (N_2_), 500 L/h; mass scan range, 100–1500 Da; and scan time, 0.25 s. Leucine enkephalin was used as the reference compound (*m/z* 554.2615 in negative ion mode). The gradient elution program comprised: 0 min, 10% B; 0–1.0 min, 10% B; 1.0–12.0 min, 10–100% B; wash for 13.4 min with 100% B; and a 1.6 min recycle time. The injection volume was 2.0 mL, and the flow rate was 0.4 mL/min.

### Cell culture

A549 and NCI-H1299 cells were purchased from the American Type Culture Collection (ATCC: Manassas, VA, USA). The human keratinocytes HaCaT cells (ATCC) were used as the control cells. The cells were cultured in RPMI 1640 medium (Welgene, Gyeongsan, South Korea) supplemented with 10% (v/v) heat-inactivated fetal bovine serum (Hyclone Laboratories, Logan, UT, USA) and maintained in an incubator at 37 °C in an atmosphere of 5% CO_2_/95% air with saturated humidity.

### Cell viability assay

Cell viability was examined by using the 3-(4,5-dimethylthiazol-2-yl)-5-(3-carboxy methoxyphenyl)-2-(4-sulfophenyl)-2H-tetrazolium (MTS) assay. The cells were seeded in 100 μL medium/well in 96-well plates (A549 cells: 0.7 × 10^4^ cells/well; NCI-H1299 cells: 0.9 × 10^4^ cells/well) and allowed to grow overnight. After 24 h, different concentrations of CG extract were added, and the cells were returned to the incubator for a further 24 or 48 h. Subsequently, the medium (100 μL) was removed and incubated with 100 μL MTS with PMS mix solution for 40 min to 1 h at 37 °C. The optical density at 492 nm was measured for each well by using an ELISA reader Apollo LB 9110 (Berthold Technologies GmbH, Zug, Switzerland).

### Annexin V/PI staining

A549 cells (1.5 × 10^5^ cells) and NCI-H1299 cells (2.0 × 10^5^ cells) were seeded in 1.5 mL medium/well in 6-well plates overnight. The cells were treated with various concentrations of CG extract for 48 h, harvested using trypsin, and washed with PBS. Annexin V and PI staining were performed by using FITC-Annexin V Apoptosis Detection Kit I (BD Biosciences, San Jose, CA, USA) in accordance with the manufacturer’s instructions. The staining was analyzed by flow cytometry using a FACSCalibur instrument and CellQuest software (BD Biosciences, San Jose, CA, USA).

### Cell cycle analysis

The cell cycle distribution was analyzed by PI (propidium iodide) staining and flow cytometry. A549 (1.5 × 10^5^ cells) and NCI-H1299 cells (2 × 10^5^ cells) were seeded in 1.5 mL medium/well in 6-well plates for overnight growth and treated with various concentrations of CG extract. After 48 h, the cells were harvested with trypsin and fixed with 80% ethanol for > 1 h. Subsequently, the cells were washed twice with cold phosphate-buffered solution (PBS) and centrifuged. The supernatant was removed, and the pellet was re-suspended and stained in PBS containing 50 μg/mL PI and 100 μg/mL RNase A for 20 min in the dark. The staining was analyzed by flow cytometry using a FACSCalibur instrument and CellQuest software (BD Biosciences, San Jose, CA, USA) to calculate the DNA content.

### Real-time quantitative polymerase chain reaction (qPCR)

A549 cells were treated with CG for 48 h, harvested, and lysed in 1 mL easy-BLUE™ (iNtRon Biotechnology, SungNam, Korea). The RNA was isolated in accordance with the manufacturer’s instructions, and cDNA was obtained by using M-MuL V reverse transcriptase (New England Biolabs, Beverly, MA, USA). Real-time qPCR was performed using a relative quantification protocol using Rotor-Gene 6000 series software 1.7 (Qiagen, Venlo, Netherlands) and a SensiFAST™ SYBR NO-ROX Kit (BIOLINE, London, UK). The expression of all target genes was normalized to that of the housekeeping gene glyceraldehyde-3-phosphate dehydrogenase, GAPDH. Each sample contained one of the following primer sets: *Fas* F: 5′-CGGACCCAGAATACCAAGTG-3′ and R: 5′-GCCACC CCAAGTTAGATCTG-3′; *FasL* F: 5′-GGGGATGTT TCAGCTCTTCC-3′ and R: 5′-GTGGCCTAT TTG CTT CTCCA-3′; *DR5* F: 5′-CACCTTGTACACGATGC TGA-3′ and R: 5′-GCTCAACAA GTGGTCCTCAA-3′; *FADD* F: 5′-GGGGAAAGATTGGAGAAGGC-3′ and R: 5′-CAGTTCTCAGTGACTCCCG-3′; *SOD2* F: 5′-TATAGAAAGCCGAGTGTTTCCC-3′ and R: 5′-GGGATGCCTTTCTAGTCC TATTC-3′; *Catalase* F: 5′-GGGATCTTTTAACGCCATT-3′ and R: 5′-CCAGTTTACCAA CTGGATG-3′; *Thioredoxin* F: 5′-GAAGCTCTG TTTGGTGCTTTG-3′ and R: 5′-CTCGAT CTGCTTCACCATCTT-3′; *GAPDH* F: 5′-GGCTG CTTTTAACTCTGGTA-3′ and R: 5′-TGG AAGATGGTGATGGGATT-3′.

### Western blotting analysis

A549 and NCI-H1299 cells were treated with CG at various concentrations for 48 h, harvested, washed with PBS, and centrifuged (13,000 rpm, 1 min, 4 °C). The cell pellets were resuspended in lysis buffer containing 50 mM Tris (pH 7.4), 1.5 M sodium chloride, 1 mM EDTA, 1% NP-40, 0.25% sodium deoxycholate, 0.1% sodium dodecyl sulfate (SDS), and a protease inhibitor cocktail. The cell lysates were mixed on a rotator at 4 °C for 1 h and clarified by centrifugation at 13,000 rpm for 30 min at 4 °C. The protein content was estimated by using a Bradford assay (Bio-Rad Laboratories, Hercules, CA, USA) and UV spectrophotometer. The cell lysates were loaded onto a 10–12% gel, separated by SDS-polyacrylamide gel electrophoresis (PAGE), and the protein bands were transferred to a polyvinylidene difluoride (PVDF) membrane (Millipore, Billerica, MA, USA). Next, the membranes were blocked with Tris-buffered saline containing Tween-20 (TBST) (2.7 M NaCl, 1 M Tris-HCl, 53.65 mM KCl, and 0.1% Tween-20, pH 7.4) and 5% skim milk for 30 min at room temperature. The membranes were incubated overnight at 4 °C with primary antibodies targeting specific proteins. After three washes with TBST for 10 min each, the membranes were incubated with a secondary antibody (HRP-conjugated anti-rabbit or anti-mouse IgG) for 2 h at room temperature. After three washes with TBST, the blots were analyzed by using a chemiluminescence detection kit (Advanstar, Cleveland, OH, USA). Western blotting bands were quantified by using ImageJ software version 1.5 [22]. The respective band intensities were normalized to GAPDH.

### Mitochondria/cytosol fractionation

A549 and NCI-H1299 cells treated with CG were collected and fractionated by using the Mitochondria/cytosol fractionation kit (BioVision Inc., San Francisco, CA, USA) in accordance with the manufacturer’s instructions. The treated cells were harvested with trypsin-EDTA and centrifuged at 600×*g* for 5 min at 4 °C. The cell pellets were suspended in 1 mL cytosol extraction reagent. The suspensions were incubated on ice for 10 min, homogenized in a sonicator, and centrifuged at 16,000×*g* for 10 min at 4 °C. The supernatant was isolated and centrifuged again at 10,000×*g* for 30 min at 4 °C; the resulting supernatant, constituting the cytosolic fraction, was transferred to a pre-chilled tube. The resulting pellet, constituting the mitochondrial fraction, was used in subsequent experiments.

### Analysis of mitochondrial membrane potential (MMP)

We evaluated MMP (Δψm) by JC-1 staining and flow cytometry. A549 (3.8 × 10^5^ cells) and NCI-H1299 (4.3 × 10^5^ cells) cells were seeded into 3 mL medium in a 60-mm culture dish and treated with various concentrations of CG. The cells were harvested with trypsin-EDTA and transferred into 1.5 mL tubes. JC-1 (5 μg/mL) was added to the cells and mixed until it was completely dissolved. Subsequently, the cells were incubated in the dark for 10 min at 37 °C, centrifuged (300×*g*, 5 min, 4 °C), washed twice with PBS, and resuspended in 200 μL PBS. The solutions were protected from light and analyzed by using a FACSCalibur instrument and CellQuest software (BD Biosciences, San Jose, CA, USA).

### Detection of intracellular ROS levels

We used a DCF-DA cellular ROS detection assay kit (Abcam, UK) to detect the accumulation of intracellular ROS in A549 and NCI-H1299 cells. A549 (0.7 × 10^4^ cells) and NCI-H1299 (0.9 × 10^4^ cells) cells were seeded into 96-well plates and incubated for 24 h in the dark. The cells were then stained with 25 μM DCF-DA for 45 min and treated with various concentrations of CG (0, 3.75, 7.5, and 15 μg/mL) for 48 h. The mean fluorescence intensity (MFI) of each well was quantified by using a fluorescence microplate reader (Gemini EM, Molecular Devices, USA) at the excitation and emission wavelengths of 485 and 538 nm, respectively.

### Statistical analysis

The data are presented as mean ± standard error of the mean (SEM), with all experiments repeated at least three times. One-way ANOVA with Tukey’s HSD test was used to analyze the significance of differences between the CG-treated groups and the untreated control group. A *p*-value of less than 0.05 was considered statistically significant.

## Results

### Identification of phytochemicals in CG extract

**Fig. 1:**
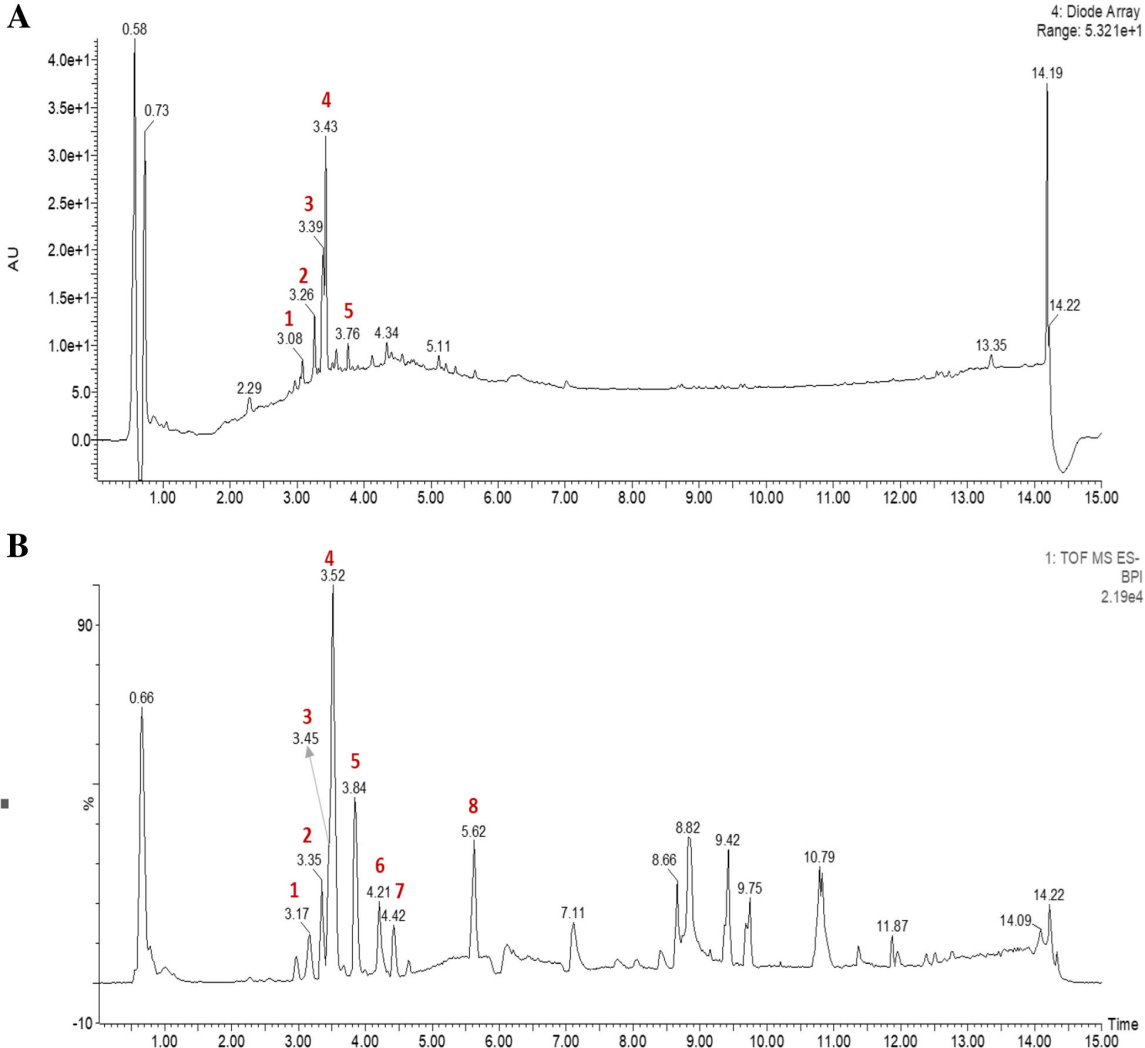
Representative mass spectrometry chromatograms of the methanol extracts of CG. **a** Diode array HPLC-MS analysis.**b** UPLC-QTof-MS analysis. The peak numbers in (**b**) are refered to Table 1

UPLC-PDA-QTof-MS analyses were performed by using a C18 column with a linear gradient of acetonitrile/water. All peaks were characterized by using mass (Fig. 1). Presented in Table 1 are the retention times, UV-Vis absorption maxima, and mass spectral data of the molecular ions of compounds in the CG extract: quercetin 3-rutinoside, kaempferol-4′-O-rutinoside, kaempferol-3-O-rutinoside, isorhamnetin-3-O-rutinoside, deglucoerycordin, 15ß-hydroxycalo tropin, frugoside, and trihydroxyoctadecenoic acid. Various rutinosides were found in CG extract and isorhamnetin-3-O-rutinoside, one of the phytochemicals found in this experiment, has been reported to have anti-cancer effects [23].

**Table 1:** Quantitative HPLC analyses of composition in CG extraction

Peak	ESI-MS RT (min)	UV (nm)	Detected ion (m/z)	Calculated ion (m/z)	Error (ppm)	MS-MS ions (m/z)	Tentative identification	Molecular formula
1	3.17	208, 254, 352	609.1424	609.1456	−5.3	179, 300, 301	Quercetin 3-rutinoside	C_27_H_30_O_6_
2	3.35	208, 265, 344	593.1505	593.1506	−0.2	179, 284	Kaempferol-4′-*O*-rutinoside	C_27_H_30_O_15_
3	3.45	203, 254, 352	593.1472	593.1506	−5.7	151, 179, 285	Kaempferol-3-O-rutinoside	C_27_H_30_O_15_
4	3.52	203, 254, 353	623.1595	623.1612	−2.7	179, 315, 593	Isorhamnetin-3-O-rutinoside	C_28_H_32_O_16_
5	3.84	219	697.3453	697.3470	−2.4	389, 535	Deglucoerycordin	C_35_H_54_O_14_
6	4.21	225	547.2565	547.2543	4.0	179, 343, 401, 419	15*b*-hydroxycalotropin	C_28_H_40_O_10_
7	4.42	225	535.2933	535.2907	0.7	179, 389	Frugoside	C_29_H_44_O_5_
8	5.62	225	329.2387	329.2328	5.9	171, 206, 211	Trihydroxy octadecenoic acid	C_18_H_34_O_5_

### CG has cytotoxic effects in A549 and NCI-H1299 cells

**Fig. 2:**
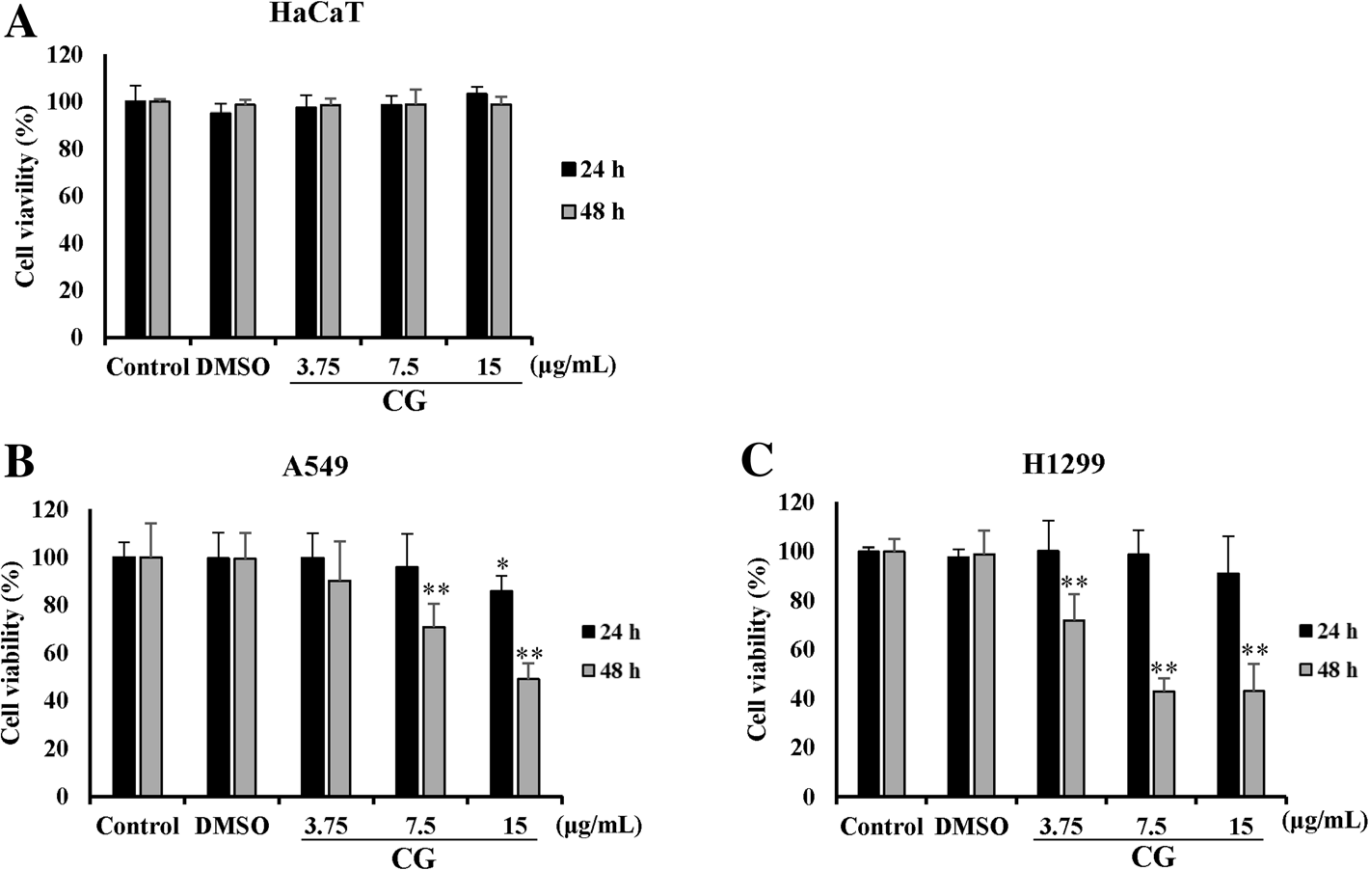
Cytotoxic effects of CG extract on A549 and NCI-H1299 NSCLC cells. The viabilities of HaCaT (**a**), A549 (**b**), and NCI-H1299 (**c**) cells. HaCaT, A549, and NCI-H1299 cells were treated for 24 h or 48 h with CG extract. The untreated cells were compared with CG-treated cells. The viability was analyzed by MTS assay. The data are presented as the mean ± SEM (*n* = 3). The data were analyzed using one-way ANOVA with Tukey’s HSD test. *, *p* < 0.05 and **, *p* < 0.005

The cytotoxic effect of CG on HaCaT, A549, and NCI-H1299 cells was determined by using an MTS assay. Three cell lines were treated with different concentrations of CG for different time periods (up to 15 μg/mL for 24 and 48 h). The viability of A549 and NCI-H1299 cells decreased in a dose-dependent manner after CG treatment (Fig. 2b and c), but that of HaCaT human normal keratinocytes was not affected by CG (Fig. 2a), confirming that CG extract exerted cytotoxic effects in A549 and NCI-H1299 human non-small cell lung cancer (NSCLC) cells only. For the positive control sample, A549 and NCI-H1299 cells were treated with doxorubicin, a chemotherapy drug. Similarly, doxorubicin decreased the viability of A549 and NCI-H1299 cells in a dose-dependent manner (Additional file 1. Figure S2). Thus, we focused our subsequent experiments to verify the mechanism through which the CG-induced apoptosis occurred in A549 and NCI-H1299 cells.

### CG induces apoptosis in A549 and NCI-H1299 cells

**Fig. 3:**
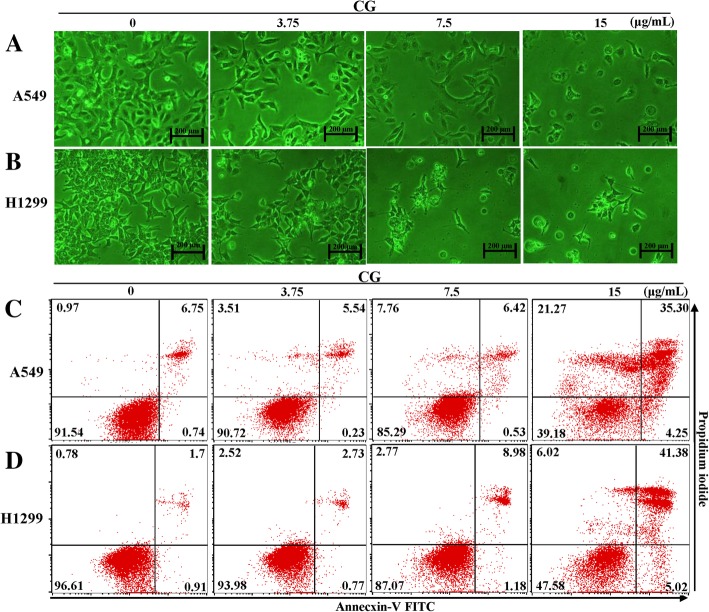
Effects of CG on viability and apoptosis in A549 and NCI-H1299 cells. Microscopic images of A549 (**a**) and NCI-H1299 (**b**) cells treated with CG for 48 h. After treatment with the indicated concentrations of CG for 48 h, A549 (**c**) and NCI-H1299 (**d**) cells were stained with Annexin V-FITC/PI. The untreated cells were compared with CG-treated cells

As the viabilities of A549 and NCI-H1299 cells for 48 h treated with CG were decreased in a dose-dependent manner, the changes in cell morphology and cell death were observed by using phase-contrast microscopy. Cell morphologies became more rounded and interacted less with surrounding cells after treatment with high concentrations of CG in A549 (Fig. 3a) and NCI-H1299 (Fig. 3b) cells than in the untreated A549 and NCI-H1299 cells. This indicated that CG could alter cell morphology and subsequently induce cell death [24]. For further evidences of the effects of CG, CG treated A549 and NCI-H1299 cells were stained with Annexin V and PI [25]. When apoptosis occurs in the cells, lipid phosphatidylserine (PS) is translocated from the inner to the outer membrane of cells, a so-called “flip-flop” movement, which allows PS to be stained with Annexin V [25]. Furthermore, pores appear in the cell membranes during necrosis or late apoptosis and mediate PI binding to DNA. Annexin V-FITC/PI staining indicated the occurrence of apoptosis in A549 (Fig. 3c) and NCI-H1299 (Fig. 3d) cells after treatment with CG. When both cell types were treated with CG for 48 h, the numbers of early and late apoptotic cells were dramatically increased, and the number of live cells decreased. These results indicated that the death of A549 and NCI-H1299 cells induced by CG was mediated by apoptosis.

### CG controls cell cycle progression in A549 and NCI-H1299 cells

**Fig. 4:**
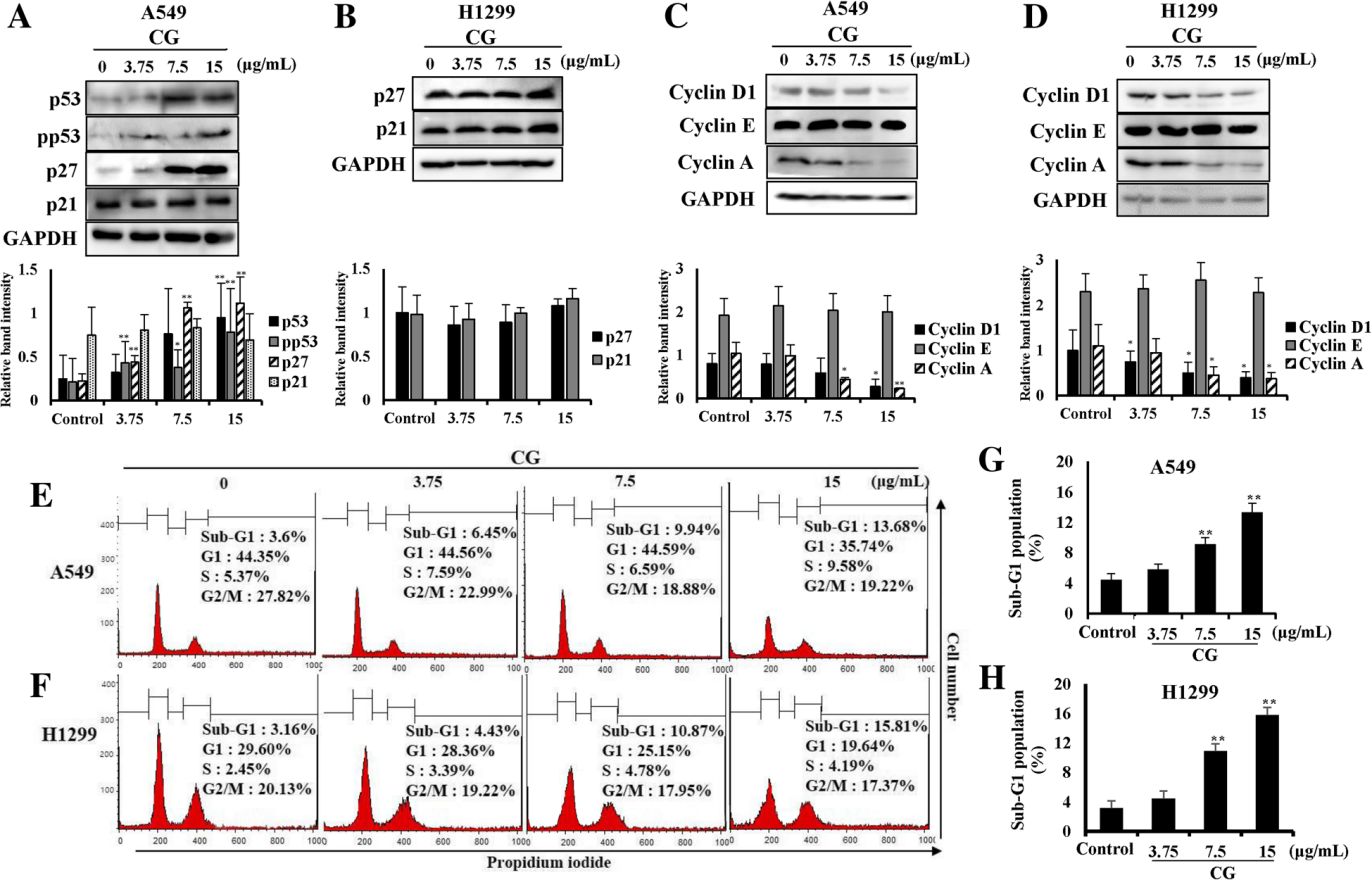
Effects of CG on the cell cycle phases in A549 and NCI-H1299 cells. **a** Protein expression of p53, p27, p21, and GAPDH in A549 cells and **b** protein expression of p27, p21, and GAPDH in NCI-H1299 cells as determined by western blotting. A549 and NCI-H1299 cells were treated with various concentrations of CG for 48 h and compared with untreated cells. The cell cycle profiles of CG-treated A549 (**c**) and NCI-H1299 (**d**) cells. The cells were treated with CG for 48 h, fixed, and stained with PI. The proportion of A549 (**e**) and NCI-H1299 (**f**) cells in the sub-G1 phase. Protein expression of cyclin D1, cyclin E, cyclin A, and GAPDH in A549 (**g**) and NCI-H1299 (**h**) cells, as determined by western blotting. The data are presented as the mean ± SEM (*n* = 3). The data were analyzed using one-way ANOVA with Tukey’s HSD test. *, *p* < 0.05 and **, *p* < 0.005

p53 is well known as a tumor suppressor protein [26] and it stimulates its downstream factor, p27 [27]. The cyclin-dependent kinase inhibitor p27 has the ability to control the cell cycle, which regulates cyclin D [28]. Proteins in the cyclin family, such as cyclins D1, E, and A, are each involved in specific phases of the cell cycle. The expression of p53 in A549 cells was increased as CG concentration increased (Fig. 4a). In addition, phosphorylated p53 (pp53; the activated form of p53), and p27 were upregulated by CG, whereas p21 was not altered (Fig. 4a). This suggested that p53 and p27 were stimulated by CG and induced the death of A549 cells through inhibiting of the cell cycle. However, in p53-null NCI-H1299 cells (Fig. 4b), p27 and p21 were not affected by CG treatment as expected. The cell cycle of CG-treated A549 (Fig. 4c) and NCI-H1299 (Fig. 4d) cells was analyzed by using flow cytometry. In the sub-G1 phase, apoptotic cells could be distinguished from fragmented DNA, which is a marker of apoptosis [29, 30]. In our study, the cell cycle analysis showed that A549 (Fig. 4e) and NCI-H1299 (Fig. 4f) cells in the sub-G1 phase increased in a dose-dependent manner by CG treatment. Furthermore, cyclin D1, especially related to the sub-G1 phase, and cyclin A were downregulated by CG treatment in A549 (Fig. 4g) and NCI-H1299 (Fig. 4h) cells, although cyclin E was not altered. These results indicated that CG extract inhibited the cell cycle of A549 and NCI-H1299 cells, by inducing the restrictions against unlimited cell growth.

### CG induces the extrinsic apoptosis pathway in A549 and NCI-H1299 cells

**Fig. 5:**
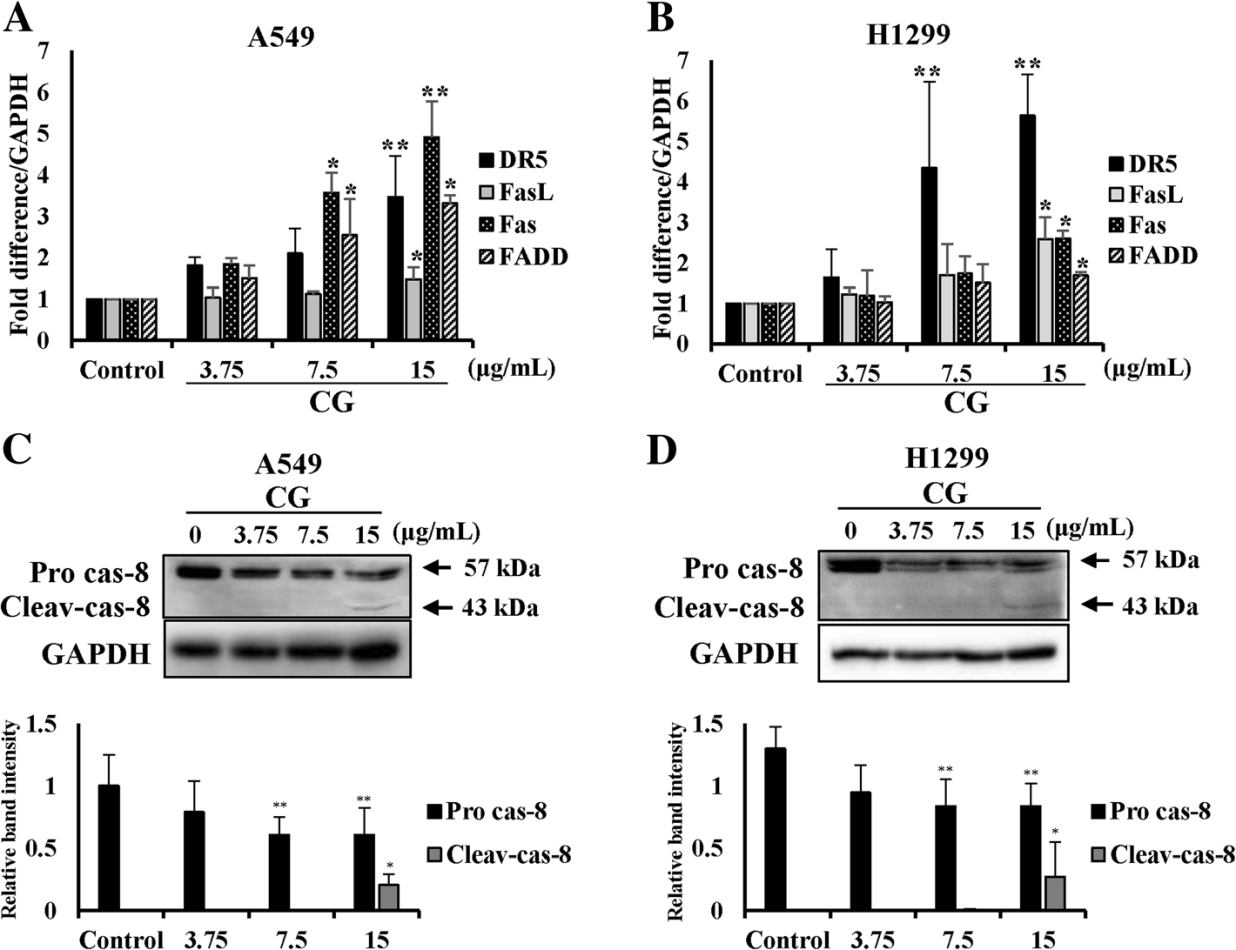
Effects of CG on extrinsic apoptosis pathway-related factors in A549 and NCI-H1299 cells. mRNA expression of DR5, FADD, Fas, and FasL in A549 (**a**) and NCI-H1299 (**b**) cells, as determined by qPCR analysis. The graph was compiled from at least three replicate analyses. Protein expression of the extrinsic pathway factors, pro-caspase-8 and its cleaved form, in A549 (**c**) and NCI-H1299 (**d**) cells, as determined by western blotting. The cells were treated with various concentrations of CG for 48 h and compared with untreated cells. The data are presented as the mean ± SEM (*n* = 3). The data were analyzed using one-way ANOVA with Tukey’s HSD test. *, *p* < 0.05 and **, *p* < 0.005. Pro cas-8, pro-caspase-8; Cleav-cas-8, cleaved caspase-8

The extrinsic apoptosis pathway is one of the main factors to leading cell death [31]. The interactions between death ligands and death receptors promote the formation of death-inducing signaling complex (DISC), which activates caspase-8 [32]. To confirm the mRNA expression of factors of the extrinsic pathway, real-time qPCR was performed. The mRNA expression of death receptor 5 (DR5), Fas-associated protein with death domain (FADD), Fas, and Fas ligand (FasL) were increased in CG-treated A549 (Fig. 5a) and NCI-H1299 (Fig. 5b) cells. Furthermore, the pro-forms of caspase-8 expression were decreased by CG in a dose-dependent manner, and the cleaved forms appeared after treatment with high concentrations of CG in A549 (Fig. 5c) and NCI-H1299 (Fig. 5d) cells. These results demonstrated that CG effectively induced cell death through the extrinsic apoptosis pathway in A549 and NCI-H1299 cells.

### CG has an apoptotic effect on mitochondrial intrinsic signaling pathways in A549 and NCI-H1299 cells

**Fig. 6:**
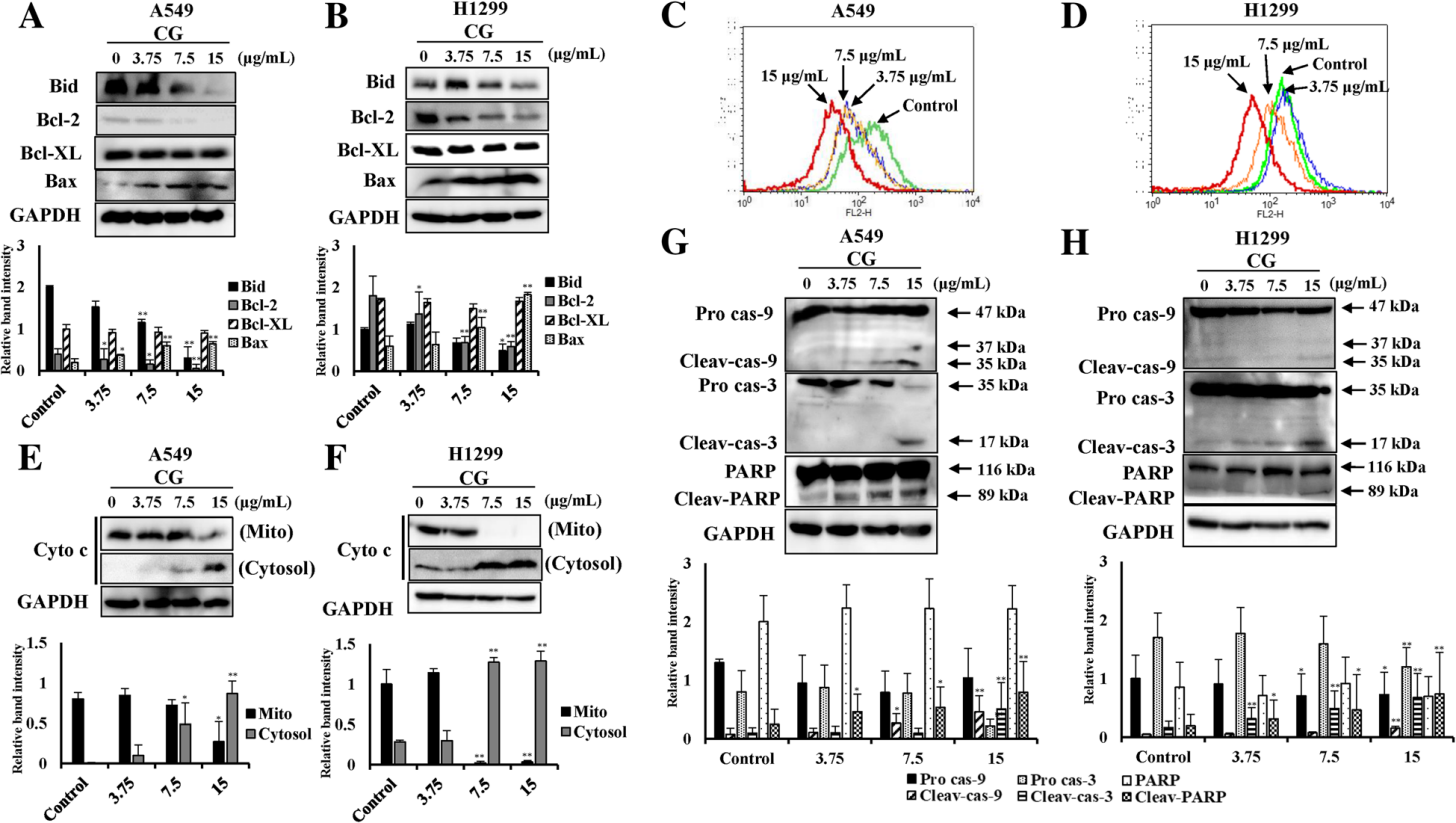
Effects of CG on MMP and intrinsic apoptosis pathway-related factors in A549 and NCI-H1299 cells. Protein expression of BID, Bcl-2, Bcl-xL, Bax, and GAPDH in A549 (**a**) and NCI-H1299 (**b**) cells, as determined by western blotting. Cells were treated with various doses of CG for 48 h and compared with untreated cells. Histogram profiles of JC-1 aggregates (FL-2, orange) detected by flow cytometry of A549 (**c**) and NCI-H1299 (**d**) cells. Western blotting of cytochrome c protein in the mitochondria and the cytosol, and GAPDH in A549 (**e**) and NCI-H1299 (**f**) cells. Protein expression of the intrinsic pathway factors, caspase-9, caspase-3, PARP, and GAPDH in A549 (G) and NCI-H1299 (H) cells, as determined by western blotting. The data are presented as the mean ± SEM (*n* = 3). The data were analyzed using one-way ANOVA with Tukey’s HSD test. *, *p* < 0.05 and **, *p* < 0.005. Cyto c, cytochrome c; Mito, mitochondria; Pro cas-9, pro-caspase-9; Cleav-cas-9, cleaved caspase-9; Pro cas-3, pro-caspase-3; Cleav-cas-3, cleaved caspase-3; Cleav-PARP, cleaved PARP

The extrinsic and intrinsic apoptotic pathways intersect in the mitochondria [33]. Activated caspase-8 cleaves the protein Bid. Cleaved Bid induces Bax-dependent outer mitochondrial membrane permeabilization and the release of cytochrome c [9]. In this study, Bid expression level was decreased, whereas Bax was enhanced in A549 cells after treatment with CG (Fig. 6a). Bcl-2, an inhibitory factor in the intrinsic apoptosis pathway, also decreased, whereas the levels of Bcl-xL were unaltered. These levels were altered in a similar manner in NCI-H1299 cells (Fig. 6b). Thus, these results suggested that MMP was decreased owing to mitochondrial dysfunction. The fluorescence of JC-1-stained cells changes from orange to green during the process of apoptosis and during a decrease in MMP. The orange fluorescence of A549 (Fig. 6c) and NCI-H1299 (Fig. 6d) cells exhibited a dose-dependent leftward shift after treatment with CG. Moreover, cytochrome c from the mitochondrial membrane appeared at high concentrations in the cytosol of CG-treated A549 (Fig. 6e) and NCI-H1299 (Fig. 6f) cells, as shown by western blotting. Mitochondrial dysfunction is a very important signal in the intrinsic pathway of apoptosis [33], and collapse of the mitochondrial membrane causes the release of caspase-9. This study confirmed these factors, such as caspase-9 and caspase-3, which are controlled by Bcl-2, were cleaved to induce apoptosis in a dose-dependent manner after CG treatment in A549 (Fig. 6g) and NCI-H1299 (Fig. 6h) cells, as determined by western blotting. The cleaved forms of caspase-9 and caspase-3 were found after treatment with the highest concentration of CG in both cells, and finally, PARP, the key element of DNA repair, was cleaved and inactivated (Fig. 6g and h). In addition, in cells treated with doxorubicin (a positive control group), PARP was cleaved to induce apoptosis (Additional file 1. Figure S3). These results indicated that CG induced apoptosis through the mitochondrial intrinsic signaling pathway in A549 and NCI-H1299 cells.

### CG generates ROS products in A549 and NCI-H1299 cells

**Fig. 7:**
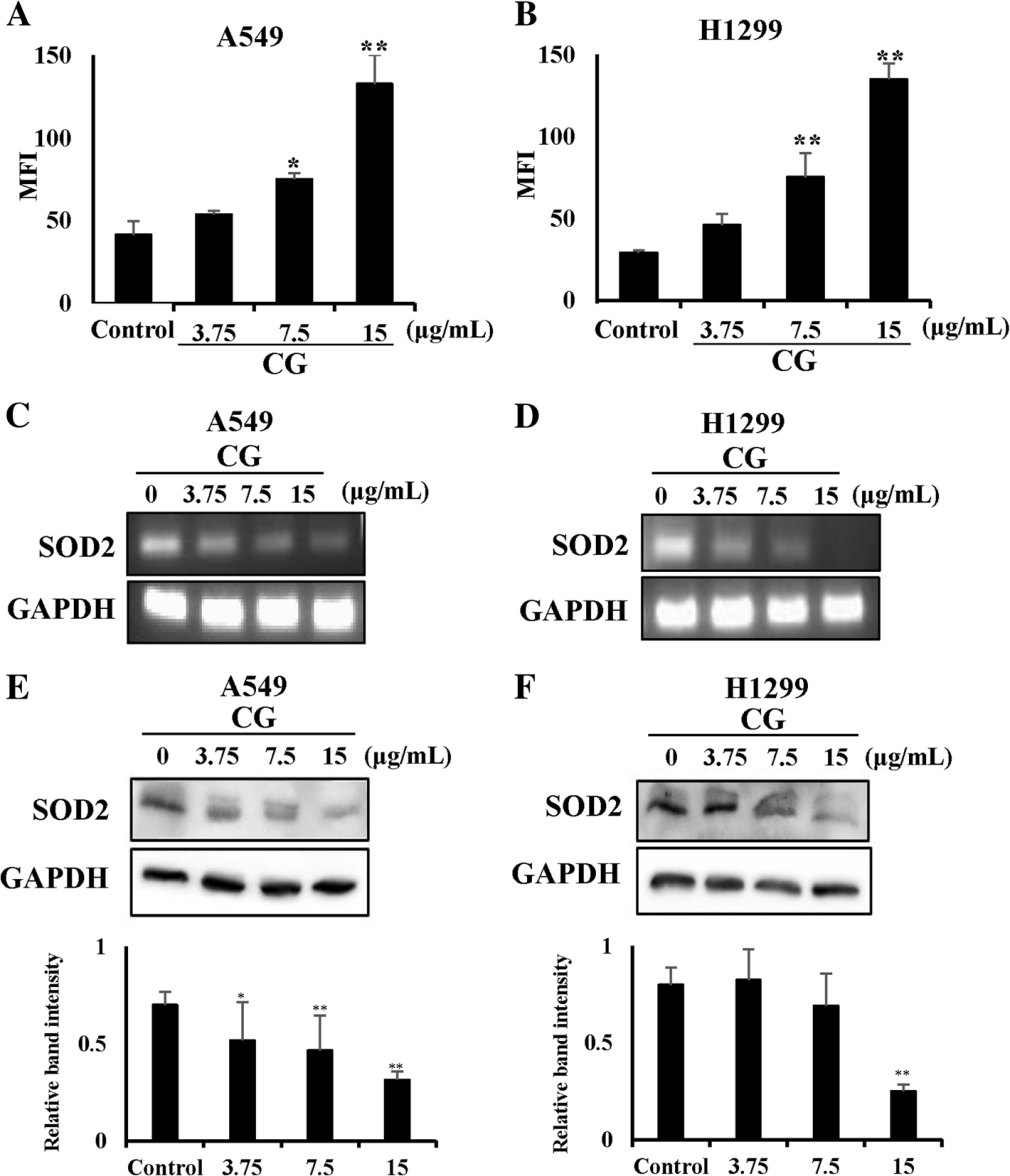
ROS generation induced by CG in A549 and NCI-H1299 cells. CG treatment resulted in ROS generation in A549 (**a**) and NCI-H1299 (**b**) cells. Cells were treated with CG for 48 h and examined using DCF-DA staining and a fluorescence microplate reader. The mRNA expression of SOD2 and GAPDH was determined by PCR analysis in A549 (**c**) and NCI-H1299 (**d**) cells treated with CG extract for 48 h. Western blots of SOD2 and GAPDH protein expression in CG-treated A549 (**e**) and NCI-H1299 (**f**) cells. The data are presented as the mean ± SEM (*n* = 3). The data were analyzed using one-way ANOVA with Tukey’s HSD test. *, *p* < 0.05 and **, *p* < 0.005

There are many studies on the relationship between ROS and apoptosis [29, 34]. We examined the generation of ROS, which is another important cause of cell death. ROS levels can be increased dramatically by environmental stress and result in significant damage, termed oxidative stress [5]. Therefore, we investigated whether CG increased ROS levels in A549 and NCI-H1299 cells. CG-treated A549 and NCI-H1299 cells produced ROS in a dose-dependent manner (Fig. 7a and b). Furthermore, the mRNA expression of the ROS scavenger, SOD2, which has an anti-apoptotic role, was decreased in a dose-dependent manner by CG treatment in A549 (Fig. 7c) and NCI-H1299 (Fig. 7d) cells and protein expression of it had a same result in both cells (Fig. 7e and f). In addition, there was a decrease in the expression of catalase, but the expression of thioredoxin (TXN) was not altered (Additional file 1. Figure S4). This study suggested that the generation of ROS mediated CG-induced apoptosis in A549 and NCI-H1299 cells.

### ROS scavenger N-acetylcysteine (NAC) restores cell viability

**Fig. 8:**
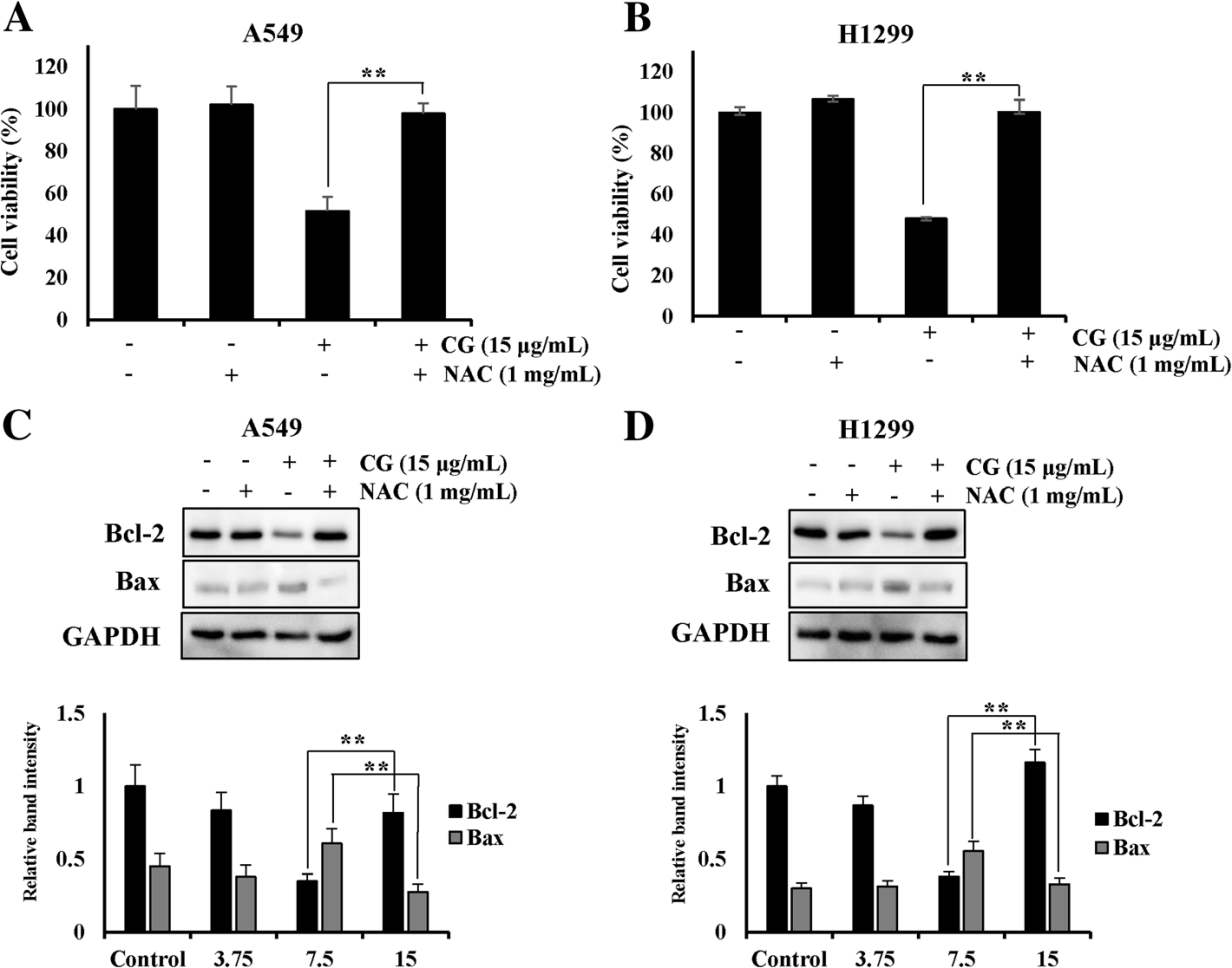
A549 and NCI-H1299 cells viability was restored by the ROS scavenger NAC. Cell viability of A549 (**a**) and NCI-H1299 (**b**) cells as determined by the MTS assay. A549 and NCI-H1299 cells were pretreated with NAC and then treated with CG for 48 h. Protein expression of Bcl-2, Bax, and GAPDH in A549 (**c**) and NCI-H1299 (**d**) cells, as determined by western blotting. The data are presented as the mean ± SEM (*n* = 3). The data were analyzed using one-way ANOVA with Tukey’s HSD test. *, *p* < 0.05 and **, *p* < 0.005

To confirm that CG extract induced apoptosis mediated by ROS generation, we used the ROS scavenger NAC [29, 35] to examine cell viability and ROS generation. In the CG/NAC-treated groups, cell viabilities were dramatically recovered to almost 100%, compared with the viability in A549 (Fig. 8a) and NCI-H1299 (Fig. 8b) cells treated with CG only. ROS levels were also decreased both in A549 and NCI-H1299 cells treated with CG and NAC (Additional file 1. Figure S5), compared with the expression in cells treated with CG only. Moreover, NAC restored the decrease in Bcl-2 and Bax after CG treatment in A549 (Fig. 8c) and NCI-H1299 (Fig. 8d) cells. Collectively, these results indicated that CG exerted anti-lung cancer effects through ROS-mediated apoptosis and that the inhibition of ROS generation by ROS scavenger NAC sufficiently blocked CG-induced apoptosis.

## Discussion

The tropical plant CG produces cardenolides, which are known anticancer compounds. The biosynthetic effects of cardenolides from CG [20] and the inhibitory effects of cardenolides on Wnt signaling, which is related to cell proliferation in colon cancer cells [21], have been reported. However, the mechanism of apoptosis induction by CG in lung cancer cells has not yet been identified. In this study, we evaluated the extent of CG-mediated apoptosis in human lung cancer cells.

First, we analyzed the chemical compounds of CG extract, and diverse rutinosides were detected (Table 1). Rutinoside is a common flavonoid that exerts anticancer effects [36]. Moreover, isorhamnetin-3-O-rutinoside, which was present at a high concentration in the CG extract, was shown to induce apoptosis in human myelogenous erythroleukemia cells [23], but had no cytotoxic effect on the NSCLC cell lines, A549 and NCI-H1299 cells (Additional file 1. Fig. S1). However, CG extract exerted cytotoxic effects on NSCLC cells, especially in A549 and NCI-H1299 cells (Fig. 2). Morphological changes in the cells were observed, and the proportion of cells in late apoptosis increased in a dose-dependent manner in CG-treated A549 and NCI-H1299 cells (Fig. 3). This indicated that the cytotoxicity of these cells was due to the apoptotic effects of CG extract.

p53 is an important tumor suppressor protein and regulates cell cycle arrest through the induction of apoptosis [37]. In this study, we confirmed that the expression of p53, phospho-p53 (pp53), and cyclin dependent kinase inhibitor p27, a downstream protein of p53, was increased in CG-treated A549 cells, but there was no alteration in their expression in CG-treated p53 null-type NCI-H1299 cells (Fig. 4a and b). During the cell cycle of A549 and NCI-H1299 cells treated with CG, the population of cells in the sub-G1 phase was increased (Fig. 4e and f). This indicated that the incidence of hypodiploid fragmented DNA in the sub-G1 phage was increased and the cell cycle was limited by CG. Cyclin D1, a key component in the activation of the sub-G1 phase of cell cycle, was marginally inhibited and cyclin A, related to DNA replication, was decreased by CG in A549 and NCI-H1299 cells (Fig. 4g and h). Collectively, the results showed that CG caused inhibitory effects in the cell cycle of A549 and NCI-H1299 cells, which stopped cell growth and induced apoptosis.

Apoptosis is the elimination of damaged cells through programmed cell death [29, 38]. We observed that CG increased the expression of the death receptors, death ligands, and the adaptors of the extrinsic pathway in A549 and NCI-H1299 cells (Fig. 5). In addition, the intrinsic pathway, through the mitochondria outer membrane permeabilization (MOMP), was induced, and cytochrome c was released from mitochondria into the cytoplasm in both types of CG-treated cells (Fig. 6). This stimulation occurred in the downstream signaling cascade that cleaved other intrinsic caspases and PARP, resulting in the induction of apoptosis by CG in both cell types (Fig. 6). Collectively, CG induced apoptosis through the stimulation of significant factors in the extrinsic and intrinsic pathway in A549 and NCI-H1299 cells.

Accumulated evidence emphasizes the principal role of ROS products that induce cell death in various cancer cell types [12, 29]. Recent studies have revealed that anticancer agents mediate their apoptotic effects through ROS [39] and that the generation of ROS products is prevented by ROS scavengers, which results in the blocking of cell death [35]. In these studies, we observed that CG enhanced ROS generation and reduced the expression of ROS scavenger genes, such as SOD2 and catalase, in A549 and NCI-H1299 cells in a dose-dependent manner (Fig. 7). Furthermore, cell viabilities and ROS levels were restored after treatment with the ROS scavenger, NAC, in both cell types (Additional file 1. Figure S5). CG extract mediated ROS-related apoptosis in A549 and NCI-H1299 cells. Collectively, CG extract induced apoptosis through the stimulation of intrinsic and extrinsic signaling pathways and the induction of cell cycle arrest and ROS generation in A549 and NCI-H1299 lung cancer cells. Further in vivo experiments and pharmacokinetic analysis should be performed to support the development of CG as an alternative therapy for lung cancer.

## Conclusions

**Fig. 9:**
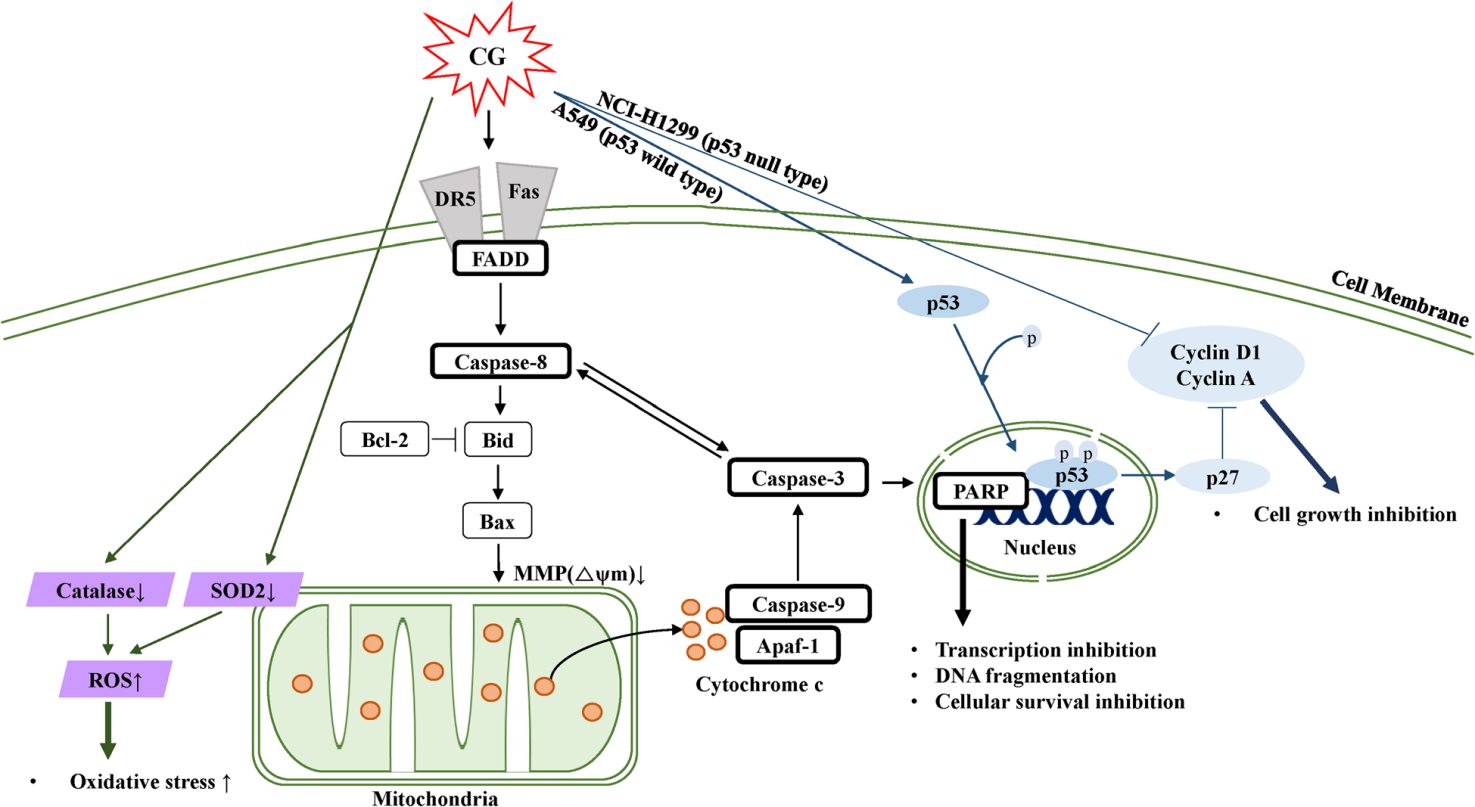
Schematic diagram illustrating CG-induced apoptotic effects in A549 and NCI-H1299 NSCLC cell lines. CG stimulated death receptor (DR5 and Fas)- and adaptor (FADD)-mediated apoptotic signaling pathways, as well as caspase-8 processing, which resulted in cytochrome c release that was regulated by Bcl-2, Bid, and Bax. Subsequently, caspase-9 and caspase-3 were activated, followed by cleaved PARP, which led to apoptosis. Furthermore, CG stimulated tumor suppressor p53, and the cell cycle was suppressed by a reduction in cyclin factors. Moreover, CG induced ROS generation through the control of ROS scavengers, such as SOD2 in mitochondria, and catalase

In conclusion, CG inhibited the proliferation of A549 and NCI-H1299 NSCLC cells via three specific mechanisms (Fig. 9). First, cell cycle arrest was induced in p53-dependent and -independent manners in A549 (p53+/+) and NCI-H1299 (p53−/−) cells, respectively. During the cell cycle, the sub-G1 population was increased through the inactivation of cyclin D1 and cyclin A in A549 and NCI-H1299 cells. Second, CG induced both the extrinsic and intrinsic apoptotic signaling pathways, which were mediated via death receptors, cytochrome c, and caspases, and this was followed by the downregulation of the DNA damage repair protein, PARP, in A549 and NCI-H1299 cells. Third, CG also produced ROS in A549 and NCI-H1299 cells, and this ROS stress led to cell death. Therefore, the CG plant extract exhibited a profound anticancer effect, and these experiments strongly support the accuracy of the proposed apoptotic mechanism of CG.

## Additional file

Additional file 1 **Figure S1.** Viability of A549 and NCI-H1299 cells treated with isorhamnetin-3-O-rutinoside. **Figure S2.** Viabilities of A549 and NCI-H1299 cells treated with different doses of doxorubicin and CG. A549 and NCI-H1299 cells were treated for 24 or 48 h with different doses of doxorubicin. **Figure S3.** Protein expression of PARP, cleaved PARP, and GAPDH in A549 and NCI-H1299 cells, as determined by western blotting**. Figure S4.** Expression of ROS scavengers in CG-treated A549 and NCI-H1299 cells. **Figure S5.** ROS scavenger N-acetylcysteine (NAC) attenuated ROS production in CG-treated A549 and NCI-H1299 cells (DOCX 541 kb)

## Data Availability

All data and materials in this study are available from the corresponding author on reasonable request.

## Abbreviations

Bax: Bcl-2-associated X protein
Bcl-2: B-cell leukemia/lymphoma 2
CG: *Calotropis gigantea*
DCF-DA: 2’,7’-dichlorofluorescin diacetate
DISC: death-inducing signaling complex
DR5: death receptor 5
FADD: Fas-associated protein with death domain
Fas L: Fas ligand
GAPDH: Glyceraldehyde 3-phosphate dehydrogenase
MMP: Mitochondrial membrane potential
MOMP: Mitochondria outer membrane permeabilization
NAC: N-acetylcysteine
NSCLC: non-small cell lung cancer
PARP: Poly (ADP-ribose) polymerase
PI: Propidium iodide
pp53: Phospho-p53
PS: Phosphatidylserine
ROS: Reactive oxygen species
SCLCs: Small cell lung cancers
SOD2: Superoxide dismutase 2
TXN: Thioredoxin
