# Supplementary material for: Calotropis gigantea extract induces apoptosis through extrinsic/intrinsic pathways and reactive oxygen species generation in A549 and NCI-H1299 non-small cell lung cancer cells
Source: BMC Complement Altern Med. 2019 Jun 18;19:134. doi: 10.1186/s12906-019-2561-1 (PMC6582476; doi:10.1186/s12906-019-2561-1)
Supplement: Supplementary file 1 — Figure S1. Viability of A549 and NCI-H1299 cells treated with isorhamnetin-3-O-rutinoside. Figure S2. Viabilities of A549 and NCI-H1299 cells treated with different doses of doxorubicin and CG. A549 and NCI-H1299 cells were treated for 24 or 48 h with different doses of doxorubicin. Figure S3. Protein expression of PARP, cleaved PARP, and GAPDH in A549 and NCI-H1299 cells, as determined by western blotting. Figure S4. Expression of ROS scavengers in CG-treated A549 and NCI-H1299 cells. Figure S5. ROS scavenger N-acetylcysteine (NAC) attenuated ROS production in CG-treated A549 and NCI-H1299 cells (DOCX 541 kb) [file 12906_2019_2561_MOESM1_ESM.docx]

**Additional data**

**H1299**

**A549**

**Control**

**Control**

**
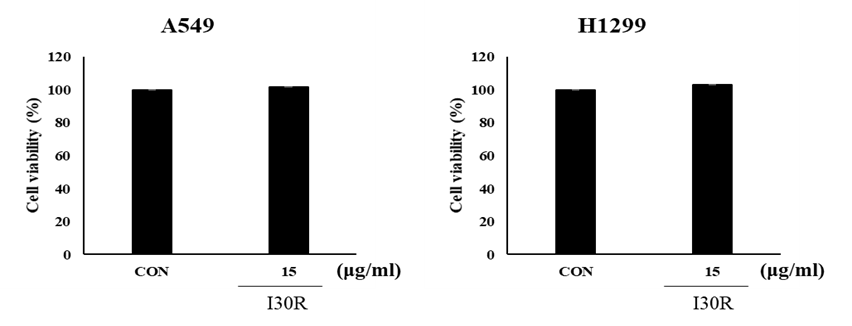
**

**Fig. S1.** Viability of A549 and NCI-H1299 cells treated with isorhamnetin-3-O-rutinoside. A549 and NCI-H1299 cells were treated with isorhamnetin-3-O-rutinoside for 48 h, and the viability was analyzed by the MTS assay.

**A)
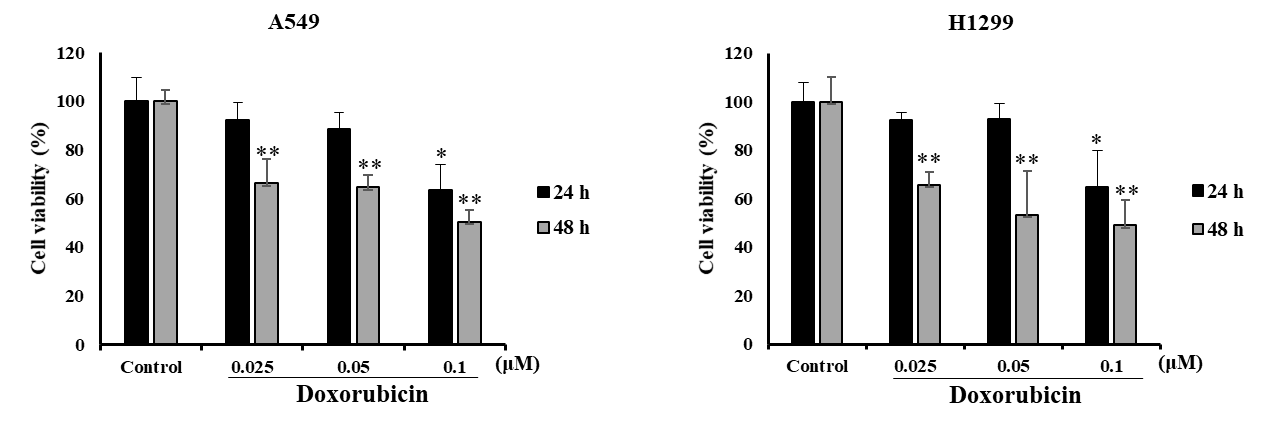
**

**B)
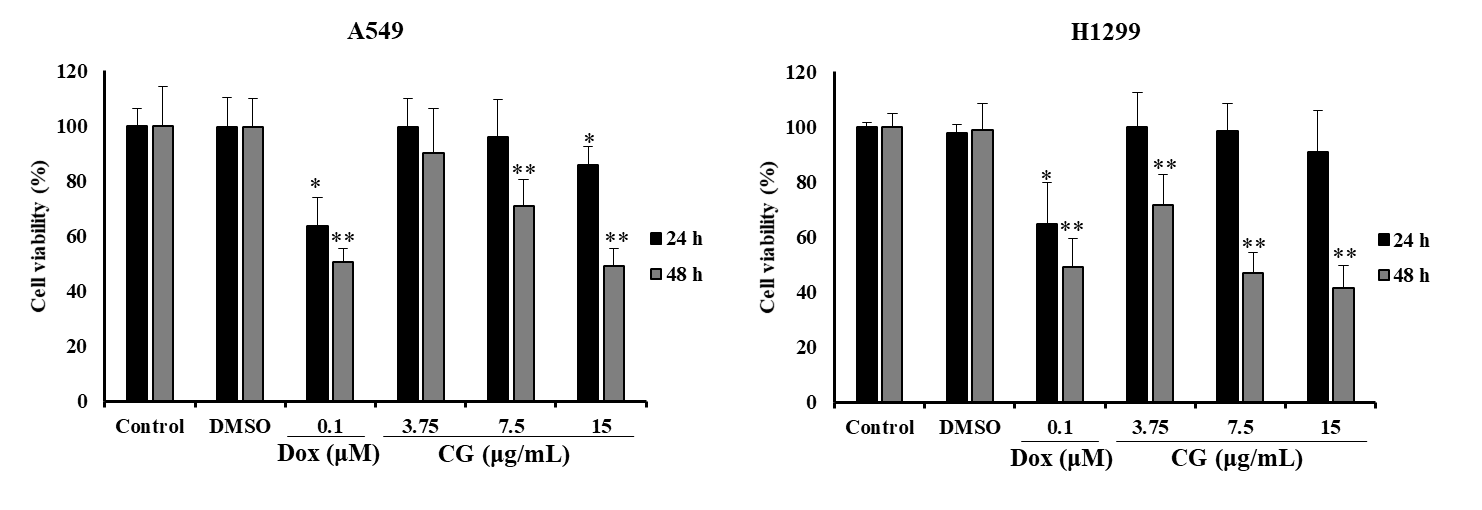
**

**Fig. S2.** Viabilities of A549 and NCI-H1299 cells treated with different doses of doxorubicin and CG. A549 and NCI-H1299 cells were treated for 24 or 48 h with different doses of doxorubicin (A). A549 and NCI-H1299 cells were treated for 24 or 48 h with doxorubicin (0.1 μM) or CG (up to 15 μg/mL) (B). Cell viability was analyzed by the MTS assay. Data are presented as the mean ± SEM (*n* = 3). The data were analyzed by one-way ANOVA with Tukey’s HSD test. *, *p* < 0.05 and **, *p* < 0.005.

**
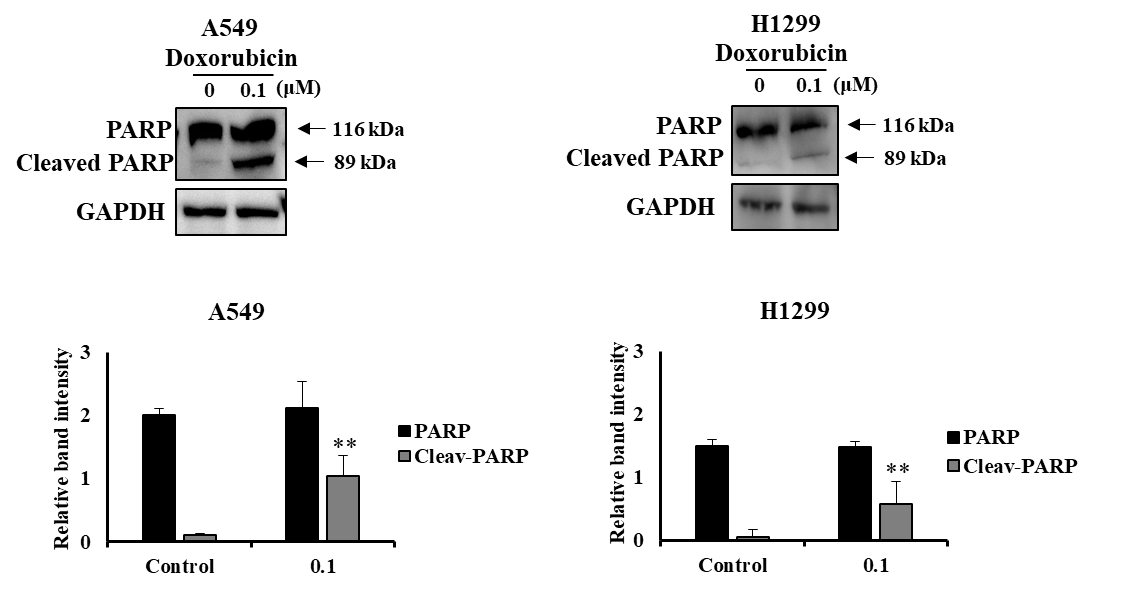
**

**Fig. S3.** Apoptotic effect of doxorubicin in A549 and NCI-H1299 cells. Protein expression of PARP, cleaved PARP, and GAPDH in A549 and NCI-H1299 cells, as determined by western blotting. The cells were treated with doxorubicin (0.1 μM) for 48 h and compared with untreated cells. Data are presented as the mean ± SEM (*n* = 3). The data were analyzed by one-way ANOVA with Tukey’s HSD test. **, *p* < 0.005.


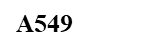

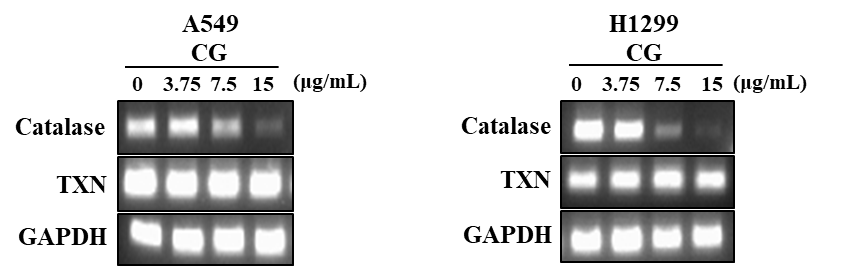


**H1299**

**Fig. S4.** Expression of ROS scavengers in CG-treated A549 and NCI-H1299 cells. The mRNA expression levels of catalase, thioredoxin (TXN), and GAPDH were determined by PCR analysis in A549 and NCI-H1299 cells treated with CG extract for 48 h.


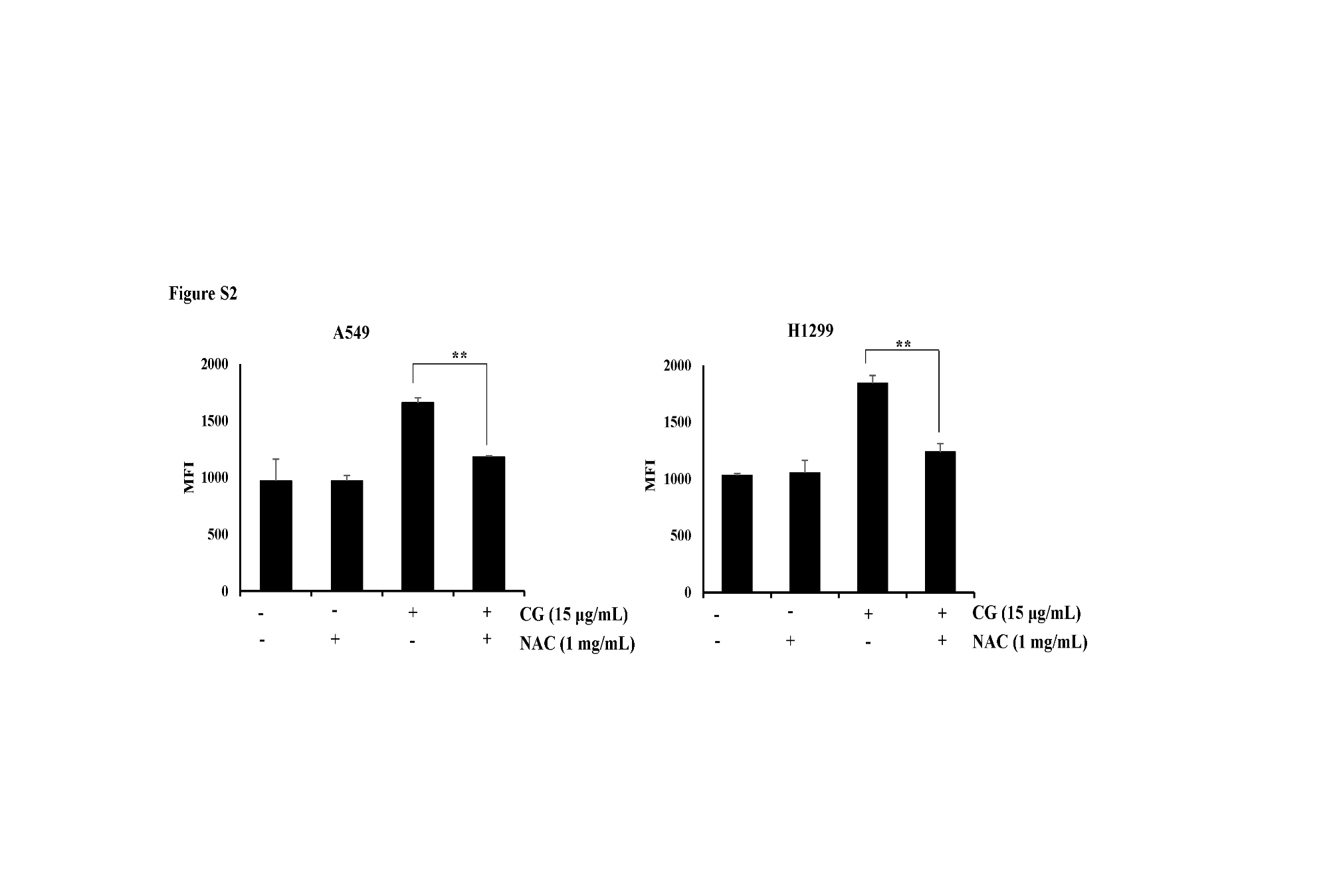


**Fig. S5.** ROS scavenger N-acetylcysteine (NAC) attenuated ROS production in CG-treated A549 and NCI-H1299 cells. A549 and NCI-H1299 cells were pretreated with NAC, treated CG for 48 h, stained with DCF-DA, and examined by using a fluorescence microplate reader. Data are presented as the mean ± SEM (*n* = 3). The data were analyzed by one-way ANOVA with Tukey’s HSD test. **, *p* < 0.005.
